# Supplementary material for: Psychosis risk among pregnant women in Ghana
Source: PLoS One. 2022 Feb 3;17(2):e0263011. doi: 10.1371/journal.pone.0263011 (PMC8812885; doi:10.1371/journal.pone.0263011)
Supplement: Attachment — Submitted filename: The study presents the results of original research.docx [file pone.0263011.s002.docx]

1. The study presents the results of original research.

Yes

2. Results reported have not been published elsewhere.

No

3. Experiments, statistics, and other analyses are performed to a high technical standard and are described in sufficient detail.

Yes

4. Conclusions are presented in an appropriate fashion and are supported by the data.

Partly

5. The article is presented in an intelligible fashion and is written in standard English.

Yes

6. The research meets all applicable standards for the ethics of experimentation and research integrity.

Yes

7. The article adheres to appropriate reporting guidelines and community standards for data availability.

Yes

To Authors

Dear authors, this is an interesting article about mental health of pregnant women. I quote below my suggestions to your manuscript.

a. Specify the exact number of the original sample and that of the final sample. It is not understood
b. The duration of the study is not mentioned in the methodology
c. How was the sample approached? Was it done during the standard check-up or during hospitalization?
d. The week of pregnancy is not specified
e. In the Introduction section describe what are the (PLEs) symptoms. In addition, for which mental disorders these symptoms are risk factors
f. Specify what the (PLEs) symptoms include in the results (eg what the anxiety symptoms include)
g. The psychometric scales are not presented in a table
h. The conclusions are not supported by the results

**Please reconsider your definition(s) in the article**

Example: Psychotic-like experiences (PLEs) are subtle, subclinical hallucinations and delusions which are quite common in general population*

**Please reconsider your conclusion in the article**

Example: The risk of developing a severe mental illness in pregnancy is estimated to be 7.1 in 10,000 per year. New-onset acute psychosis during pregnancy is extremely rare**

*Remberk B. Clinical significance of psychotic-like experiences in children and adolescents. Psychiatr Pol 2017, 51:271-82.

**Watkins ME, Newport  J. Psychosis in Pregnancy. Obstetrics & Gynecology 2009, 113: 1349.
