# Supplementary material for: Psychosis risk among pregnant women in Ghana
Source: PLoS One. 2022 Feb 3;17(2):e0263011. doi: 10.1371/journal.pone.0263011 (PMC8812885; doi:10.1371/journal.pone.0263011)
Supplement: Attachment — Submitted filename: Response to Reviewer comments.docx [file pone.0263011.s003.docx]

Psychosis Risk among Pregnant Women in Ghana

PONE-D-21-20182

RESPONSE FROM AUTHORS: We have noted our responses to the reviewers’ comments below in yellow highlight. We appreciate the feedback from the reviewers.

**Reviewer 1 Comments**

Dear authors, this is an interesting article about mental health of pregnant women. I quote below my suggestions to your manuscript.

a. Specify the exact number of the original sample and that of the final sample. It is not understood.

**Authors’ response**: A total of 702 women were recruited for the study but some were missing data on some study variables. Excluding participants with missing data in the analyses caused a reduction in the sample size across.

b. The duration of the study is not mentioned in the methodology

**Authors’ response**: This has been specified as follows: “Data were collected from September to October 2020”

c. How was the sample approached? Was it done during the standard check-up or during hospitalization?

**Authors’ response**: We have included this information in the revised manuscript as follows: “The participants, who often congregate at the antenatal clinics of the facilities for their antenatal services, were informed about the purpose and duration of the study, their responsibilities for participating in the study, ethical issues such as confidentiality, consent, anonymity and benefits of participation.” (page 6).

d. The week of pregnancy is not specified

**Authors’ response**: We included data on the weeks of pregnancy as follows: “Pregnant women in their first (≤ 12 weeks of pregnancy), second (≤ 24 weeks of pregnancy) or third trimesters (≤ 36 weeks of pregnancy) were recruited for the study.” (page 6).

e. In the Introduction section describe what are the (PLEs) symptoms. In addition, for which mental disorders these symptoms are risk factors

**Authors’ response**: A brief description of PLEs is provided. The section now reads:

“PLEs are very common in the general population, appearing first in adolescence and sometimes in childhood (Zavos et al., 2014) and can be categorized as positive (e.g., perceptual abnormalities, delusional thoughts) or negative (e.g., social withdrawal, avolition) (Yung et al., 2009). Among the common examples of the PLEs are hearing voices, seeing things, and smelling things that other people do not hear, see or smell, respectively.” (page 4).

f. The psychometric scales are not presented in a table

**Authors’ response**: In table 2, we provided data on the Cronbach alpha for all the scales used in the study.

g. The conclusions are not supported by the results

**Authors’ response**: We have revised the conclusion section of the manuscript accordingly.

**Please reconsider your definition(s) in the article**

Example: Psychotic-like experiences (PLEs) are subtle, subclinical hallucinations and delusions which are quite common in general population*

**Authors’ response**: We have expanded on the initial definition provided in the manuscript. The section now reads:

“PLEs are subclinical symptoms of psychosis that do not meet the threshold for clinical diagnosis as psychotic illness (Fusar-Poli et al., 2012; Hielscher et al., 2018). PLEs are very common in the general population, appearing first in adolescence and sometimes in childhood (Zavos et al., 2014) and can be categorized as positive (e.g., perceptual abnormalities, delusional thoughts) or negative (e.g., social withdrawal, avolition) (Yung et al., 2009). Among the common examples of the PLEs are hearing voices, seeing things, and smelling things that other people do not hear, see or smell, respectively.” (page 4).

*Remberk B. Clinical significance of psychotic-like experiences in children and adolescents. Psychiatr Pol 2017, 51:271-82.

 **Watkins ME, Newport  J. Psychosis in Pregnancy. Obstetrics & Gynecology 2009, 113: 1349.

**Authors’ response**: Thank you very much for these articles. They are extremely helpful.

Reviewer #2: This paper uses a cross-sectional design to investigate risk factors during pregnancy for postpartum psychosis (PPP). It adds to an important area of the perinatal mental health literature, as PPP is the most severe mental illness in the perinatal period and not many studies have investigated psychotic-like experiences during pregnancy as a risk factor for PPP. A strength of the study is the large sample size the authors obtained data from.

I have divided my comments into major and minor points.

Major:

1. This study feels incomplete without following up with women in the postpartum period to understand who went on to develop PPP within the sample. Given that the study examined risk factors/correlates of psychotic-like experiences in pregnancy, and PLEs can be a starting point of PPP, it would be helpful to know whether those women who scored high on the PQ-16 questionnaire and had identified correlates (depression, suicidal ideation, COVID-19 anxiety, sleep disturbances) actually developed PPP symptoms in the end. This way we can understand whether 1) the identified correlates may in themselves be risk factors for PPP, and 2) the PQ-16 questionnaire is a valid predictor of PPP in itself. Furthermore, it would help the authors to determine whether a cut-off score can be used on the PQ-16 to identify pregnant women at greatest risk.
**Authors’ response**: It is true that following up on the participants after delivery to determine those diagnosed with psychosis would add significantly to the findings of the study. Our main goal in this study was to determine the prevalence of psychotic like experiences (PLEs). Individuals scoring high on PLEs are at risk for psychotic illness and not necessarily that they would develop psychotic illness. Therefore, it is possible that none of the participants would have been diagnosed with psychotic illness at follow-up. The discussion would have been different if we had screened for psychosis as a mental disorder. In view of this, we are happy to contribute to the emerging literature on the prevalence and correlates of PLEs among pregnant women in Ghana. We are optimistic that the findings will serve the research community in terms of opening up future research areas.

2. Please discuss why the authors chose not to include participants with a history of mental health disorder/current mental health disorder as these are one of the biggest risk factors for PPP? Especially given that it was found that main correlates of high PLE scores were depressive and anxious symptoms and suicidal ideation, it would be important to understand whether pregnant women with histories of (or current) mental illness score highly on the PQ-16.
**Authors’ response**: We initially collected data on individuals with a history of mental disorders (n = 5). However, our initial analyses reveal that they scored very high on the measures of anxiety and depression etc, emerging as outliers. To maintain sample purity, we agreed to exclude them. If their numbers were high, we could have compare them with those without diagnoses of mental disorders.

3. With regard to the questions on suicidal ideation and intimate partner violence, please state whether a factor analysis was completed to determine that these items are valid extractions of an entire questionnaire.

**Authors response**: Thank you for the observation. Yes, we subjected the items measuring suicidal ideation and IPV to principal component analysis. We requested for one component given the small number of items. All the items loaded satisfactory on the component, with correlation coefficients ranging from .52 to.89. Based on PCA, we treated the added the items measuring suicidal ideation and IPV to obtain unidimensional scores that were used for the analyses

4. Given that there was multicollinearity between many of the variables in Table 2, they should not have all been entered into a regression together.
**Authors’ response**: We observed a correlation of .71 between anxiety and depressive symptoms. Other correlations ranged from .19 to 41, which are normal for regression analysis. The correlation coefficient of .71 appear high but there was no evidence of multicollinearity in the regression analysis.

Minor:

1. In the introduction, please discuss PPP prevalence across more societies/cultures than only the US and Sweden, as both are high-income countries.

**Authors’ response**: Thank you for the observation. Unfortunately, data is limited from other context. In a South African we have referenced in the revised manuscript, there was no information on prevalence. Rather, data were collected from individuals diagnosed with PPP. We have noted this in the revised manuscript (page 3).

2. Please discuss whether the PQ-16 has been previously validated as a predictor of PPP in a perinatal sample, rather than a predictor of psychosis in a general sample.

**Authors’ response**: The PQ-16 has been validated to screen for psychotic-like experiences in the general population (Ising et al., 2012) and pregnant women (Levey et al., 2018).

3. Related to point 2, please discuss whether the PQ-16 has been validated across various cultures and ethnicities.

Authors’ response: With respect to pregnant women, the PQ-16 has been validated in Peruvian pregnant women (Levey et al., 2018) only.

4. Please reference Hazelgrove et al. (2021)’s paper on risk factors for postpartum psychosis.

**Authors’ response**: Thank you very much. The reference has been included in the revised manuscript.

5. Table 1: Please indicate whether the values are listed as number (%) or %(number).

**Authors’ response**: We have indicated in the revised manuscript that the figures in the bracket represent percentages.

6. In addition to asking women whether they were concerned about COVID-19, were they also asked whether they had contracted the virus? This is especially important as COVID-19 has been found to cause psychotic-like symptoms in some patients.

**Authors’ response**: We did not ask the participants whether they contracted the virus. This is an excellent observation that would be incorporated into our future work.
