# Supplementary material for: Psychosis risk among pregnant women in Ghana
Source: PLoS One. 2022 Feb 3;17(2):e0263011. doi: 10.1371/journal.pone.0263011 (PMC8812885; doi:10.1371/journal.pone.0263011)
Supplement: Attachment — Submitted filename: Response to reviewer comments 2.docx [file pone.0263011.s004.docx]

Psychosis Risk among Pregnant Women in Ghana

PONE-D-21-20182R1

RESPONSE FROM AUTHORS: We have noted our responses to the reviewers’ comments below in yellow highlight. We appreciate the feedback from the reviewers.

**Reviewer Comment 1**: Abstract: Perinatal psychosis is a very rare and not a common illness
Response: The statement has been revised as follows;

“Psychotic illness, although is rare, has been reported in the perinatal period.”

**Reviewer Comment 2:** Introduction: It is important to mention at this point the risk factors for developing psychosis, such as bipolar disorder, family history, atomic history of psychotic episode etc.
Authors’ response: Thank you. We have included in the manuscript risk factors culled from the perinatal factors as follows;

As a heterogeneous mental disorder, several risk factors have been documented, including childhood maltreatment (Hazelgrove et al., 2021), low socio-economic status, neighborhood level social deprivation (Radua et al., 2018). Among the perinatal factors implicated in psychosis include diabetes in pregnancy, antepartum haemorrhage, preeclampsia, maternal stress during pregnancy, pre-pregnancy and pregnancy maternal obesity and cord complications (Radua et al., 2018).

**Reviewer Comment 3**: It would be better to summarize the psychometric tools

Authors’ response: Thank you very much. We are not what you meant by summarize the psychometric tools. This study is not a validation study and so detailed psychometric properties was not presented. As much as possible, we presented the reliability of the questionnaires that are not categorical. This was done for each measure as far as practicable.

**Reviewer Comment 4**: As for Covid, is there any information on whether women were exposed to the virus or not?

Authors’ response: Thank you. We are not aware of the exposure status of the women who took part in the study. We can only deduce that since they were not recruited from isolation centers, or their medical records did not contain evidence of COVID-19, they probably did not contract COVID-19.

**Reviewer Comment 5:** In the conclusions, what interventions do you suggest to perinatal health care professionals regarding the early recognition of psychotic symptoms? What do you suggest to improve this situation?

Authors’ response: Thank you very much. We noted in the manuscript that this is one of the initial studies exploring psychotic-like-experiences in pregnancy. Therefore, we are careful to state the usefulness of the findings to healthcare delivery. At this stage, we can only recommend, as stated in the manuscript (conclusion section) that screening for psychotic-like experiences should be incorporated into the existing perinatal care services with the hope of picking at risk women for early intervention.

**Reviewer Comment 6**: What measures should be taken to prevent further development of the disorder in postpartum period?

Response: This is a very important question. Unfortunately, we could not follow the participants from pregnancy to post-delivery periods to know the rate of conversion to full-blown psychosis. Therefore, we could not comment on measures to prevent the development of psychosis in the postpartum period.
