# Supplementary material for: Psychosis risk among pregnant women in Ghana
Source: PLoS One. 2022 Feb 3;17(2):e0263011. doi: 10.1371/journal.pone.0263011 (PMC8812885; doi:10.1371/journal.pone.0263011)
Supplement: Attachment — Submitted filename: Reviewer comments.docx [file pone.0263011.s005.docx]

Reviewer #1: Dear Authors,
You have made enough effort to improve the article. However, some difficult points remain unclear
• With regard to the scales of measurement, I referred to the possibility of presenting them in less detail.

Authors’ response: Thank you very much for the feedback. It is our understanding that the scales of measurement have been presented in a manner that the research community can follow. We would be happy if this is accepted the way it is.

• Furthermore, even if a study is in its early stages clear conclusions from the study need to be presented. The conclusions also include the authors' suggestions as well as any interventions required.

• In addition to identifying a significant mental health problem in the postpartum period, researchers should be able to come up with some preventative measures to reduce them. For example, identifying risk factors from pregnancy or early postpartum period, educating perinatal health care professionals on recognizing dangerous symptoms, educating the family on caring for women with mental disorders and others….
Regards

Authors response: We are grateful for the comments. In the earlier version of the manuscript, we accepted your recommendation and included a conclusion statement. In this revised version, we have expanded on this conclusion section as follows;

“The study has provided initial evidence regarding the prevalence of PLEs in pregnancy in a Western Africa country, Ghana. It is envisaged that the findings will serve as a wake-up call for researchers to investigate PLEs and psychosis in the perinatal period as a public health issue. Healthcare professionals should equally consider to include PLEs screening measures into the existing gamut of mental health screening tools used in the perinatal period. Once screened, education and awareness creation on psychosis and psychosis-risk should be undertaken to increase the knowledge-base of pregnant women. Information on the characteristics, manifestation of psychotic-like symptoms, the transition to full-blown psychotic disorders and negative impacts of psychosis on pregnancy and pregnancy outcomes should be incorporated into the education and awareness program. It is recommended that future studies are conducted to unearth the nature or mechanisms as well as the risk and protective factors of the trajectory of PLEs to clinically diagnosable psychosis.”
